# Persistent air leak successfully treated with endobronchial valves and digital drainage system

**DOI:** 10.1002/rcr2.368

**Published:** 2018-09-14

**Authors:** Thomas James Altree, Hubertus Jersmann, Phan Nguyen

**Affiliations:** ^1^ Department of Thoracic Medicine Royal Adelaide Hospital Adelaide South Australia Australia

**Keywords:** Bronchoscopy, digital chest drainage system, endobronchial valve, persistent air leak, pneumothorax

## Abstract

A 62‐year old man with severe chronic obstructive pulmonary disease developed a persistent air leak from an iatrogenic pneumothorax following Computed Tomography‐guided core biopsy of a pulmonary nodule. The pneumothorax was treated with an 8.5F intercostal catheter, which was then replaced by a 28F thoracostomy tube after development of significant subcutaneous emphysema and a tension pneumothorax. The air leak showed no improvement until endobronchial valve (EBV) insertion guided by objective flow data from a digital drainage system (DDS). The air leak subsequently reduced with −20 cmH_2_O suction from the DDS, and the thoracostomy tube was removed once the objective measured flow rate had sufficiently diminished. The combination of EBV insertion and suction from the DDS successfully treated the persistent air leak, with timing of thoracostomy tube removal guided by DDS flow data.

## Introduction

Persistent air leaks (PALs) in patients with severe parenchymal lung disease pose a difficult management dilemma, with no clear consensus on the optimal treatment approach. Traditionally, PAL is defined as air leak lasting five or more days based on surgical data 1.

Bronchoscopic treatment of PAL with endobronchial valves (EBVs) has been described in several case series, but accurate localization of the leak remains a clinical challenge. Cardiothoracic surgical data have shown that digital drainage systems (DDS) are a useful tool to help guide the timing of postoperative chest drain removal 2, 3, 4. Such systems are probably underutilized in respiratory medicine for leak localization during bronchoscopy. We describe a case where EBV placement for treatment of a PAL was guided by the use of a DDS, with subsequent timing of chest tube removal also successfully predicted from DDS leak data.

## Case Report

A 62‐year old man with rheumatoid arthritis treated with tofacitinib and severe chronic obstructive pulmonary disease was admitted after developing an iatrogenic pneumothorax from a Computed Tomography‐guided core biopsy of a left upper lobe lung nodule. He remained hemodynamically stable but had dyspnoea, oxygen desaturation, and chest pain. His symptoms improved after insertion of an 8.5F intercostal catheter that was attached to an underwater seal drain (UWSD), but over the following two days, a large air leak persisted, and a chest radiograph showed a persistent small left apical pneumothorax. Histology of the nodule demonstrated necrotizing granulomas and no evidence of malignancy.

**Figure 1:**
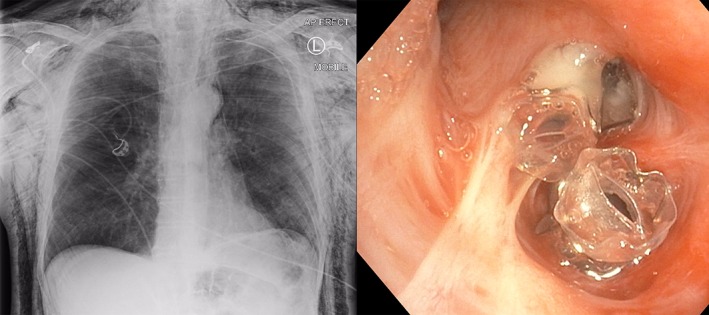
Chest radiograph after thoracostomy tube insertion demonstrating significant subcutaneous emphysema and endobronchial valves in situ in LB3 and LB4+5.

On day three of admission, the patient developed progressively worsening subcutaneous emphysema. Chest radiograph showed minor retraction of the intercostal catheter, and a sideport was thought to be located in the subcutaneous tissue. The catheter was removed, and the patient rapidly became hemodynamically unstable, with diminished left‐sided breath sounds and type 2 respiratory failure. Tension pneumothorax was diagnosed, and a 28F thoracostomy tube was inserted, attached to a UWSD (Fig. 1).

From days three to 23, the patient’s clinical state improved, but there was no reduction in air leak.

On day 23 of admission, EBV insertion was performed. Immediately prior to the procedure, the UWSD was switched to a DDS (Rocket® Portable Suction Unit (PSU), Rocket Medical, England) to provide objective flow data during bronchoscopic balloon occlusion of the left upper lobe airways. There was no reduction in flow on the PSU digital display when individual left upper lobe segmental airways were occluded with a Fogarty catheter, but there was marked reduction in flow with balloon occlusion of the entire left upper lobe bronchus. Use of the Chartis® System (Pulmonx, Switzerland) showed no evidence of left‐sided lobar collateral ventilation. Given the patient’s severe chronic obstructive pulmonary disease and diminished respiratory reserve, the decision to induce total lobar collapse with EBVs was decided against, but based on the anatomical position of the air leak caused by the CT‐guided biopsy, one Pulmonx Zephyr® 5.5 mm EBV was deployed into LB3 and another into the lingula bronchus (Fig. 1). He returned to the ward with the PSU set at −20 cmH_2_O.

On days 23–29, the flow readings showed ongoing, but steadily diminishing, air leak. The patient expressed a wish to return home, so he was discharged with the thoracostomy tube attached to a Pneumostat® Chest Drain Valve (Maquet, Germany) and a plan for elective readmission 2 weeks later (day 43) to reattach the PSU and measure the degree of leak.

**Figure 2:**
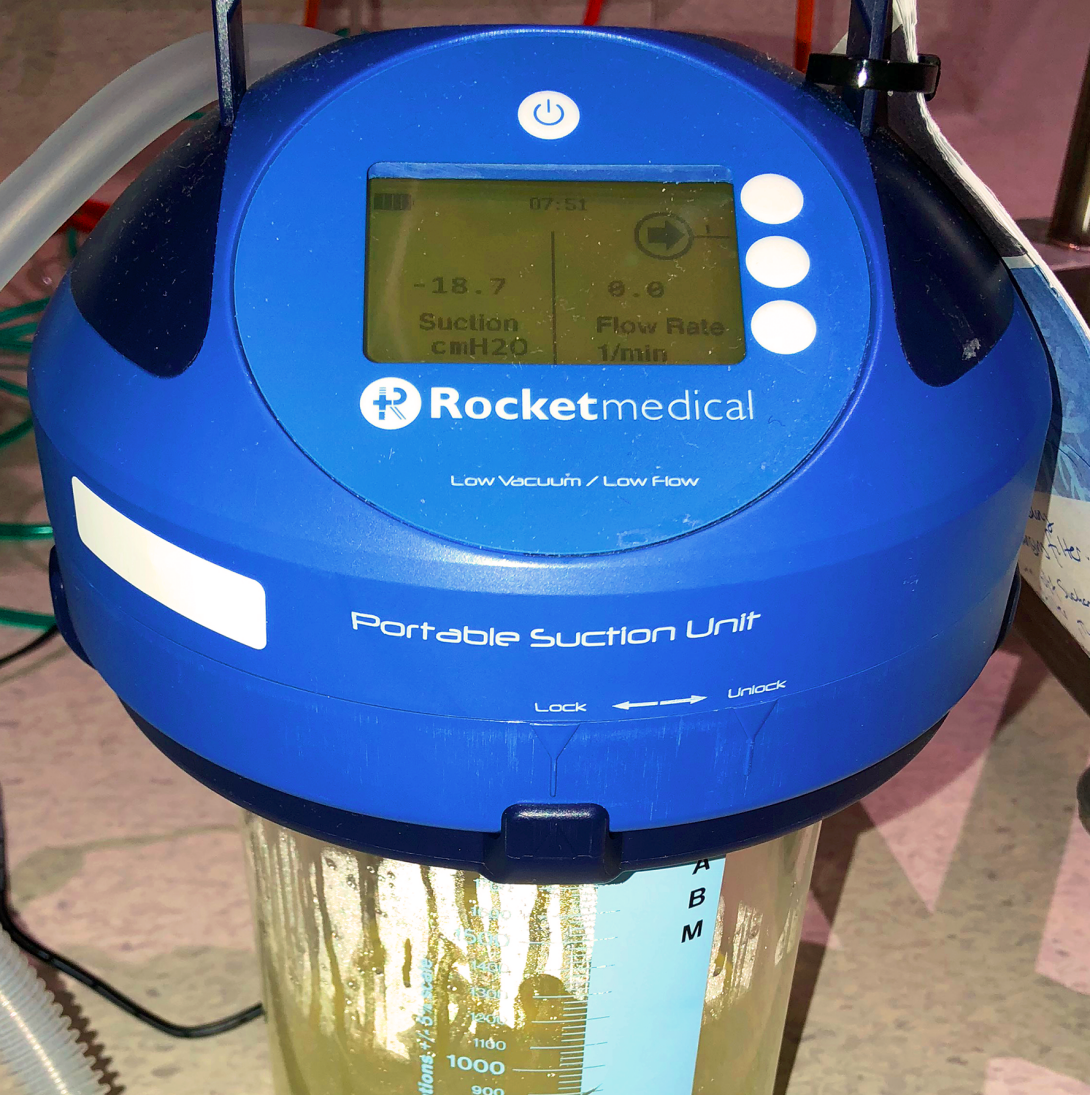
Rocket® Portable Suction Unit on suction showing no flow prior to thoracostomy tube removal.

On day 41, the patient represented with a febrile illness and mild pain and erythema at the thoracostomy tube site. A superficial, localized wound infection was diagnosed, and he was commenced on intravenous antibiotics. Occasional, but reduced, bubbling was noted in the Pneumostat® device. Chest radiograph showed no discernible pneumothorax. The Rocket® PSU was reattached on constant suction (−20 cmH_2_O). By day 43, air leak flow readings had reduced to zero, and bubbling ceased (Fig. 2). The thoracostomy tube was safely removed, with repeat imaging showing no pneumothorax. The wound infection resolved with antibiotics, and on day 46, he was discharged home.

## Discussion

The emerging use of EBVs for persistent air leak that fails conventional therapy has added an effective, minimally invasive option in what is often a difficult‐to‐treat condition. The largest case series in this area demonstrated a complete or partial air leak resolution in 93% (*n* = 37/40) of patients treated with EBVs 5. Despite insertion of a 28F thoracostomy tube, our patient’s PAL did not reduce until after EBV insertion.

The use of a DDS helped to objectively quantify the air leak during sequential bronchoscopic occlusion of the left upper lobe airways. DDSs display real‐time air leak flow rates (in mL/min), thus allowing objective measurement of flow, which informs EBV deployment site decision‐making and chest drain removal timing. This latter use has been well established postoperatively in large randomized trials but requires further study in non‐surgical cohorts 2, 3, 4.

DDSs can also provide regulated suction based on the current pleural pressure. After EBV insertion, attaching a DDS set at ‐20cmH20 led to the resolution of our patient’s PAL. The use of suction in medical (as opposed to postoperative) patients remains controversial. In this case, it was our opinion that healing of the visceral pleura was more likely to occur if the lung was fully expanded and apposed to the parietal pleura.

Our case demonstrates the beneficial role that EBVs can play in patients with persistent air leak and that the suction and objective flow data provided by DDSs aids leak healing, EBV deployment decisions, and decision‐making regarding timing of chest drain removal.

### Disclosure Statement

Appropriate written informed consent was obtained for publication of this case report and accompanying images.

## References

[1] Varela G , Jiminéz MF , Novoa NM , et al. 2005 Estimating hospital costs attributable to prolonged air leak in pulmonary lobectomy. Eur. J. Cardiothorac. Surg. 27:329–333. 15691691 10.1016/j.ejcts.2004.11.005

[2] Cerfolio RJ , and Bryant AS . 2008 The benefits of continuous and digital air leak assessment after elective pulmonary resection: a prospective study. Ann. Thorac. Surg. 86:196–401. 10.1016/j.athoracsur.2008.04.016 18640304

[3] Varela G , Jiminéz MF , Novoa NM , et al. 2009 Postoperative chest tube management: measuring air leak using an electronic device decreases variability in the clinical practice. Eur. J. Cardiothorac. Surg. 35:28–31. 18848460 10.1016/j.ejcts.2008.09.005

[4] Pompili C , Detterbeck F , Papagiannopoulos K , et al. 2014 Multicenter international randomized comparison of objective and subjective outcomes between electronic and traditional chest drainage systems. Ann. Thorac. Surg. 98:490–496. 24906602 10.1016/j.athoracsur.2014.03.043

[5] Travaline JM , McKenna RJ , Giacomo TD , et al. 2009 Treatment of persistent pulmonary air leaks using endobronchial valves. Chest 136:355–360. 19349382 10.1378/chest.08-2389

